# Therapeutic inertia in the management of neuromyelitis optica spectrum disorder

**DOI:** 10.3389/fneur.2024.1341473

**Published:** 2024-02-21

**Authors:** Álvaro Cobo-Calvo, Rocío Gómez-Ballesteros, Aida Orviz, María Díaz Sánchez, Sabas Boyero, Marta Aguado-Valcarcel, María Sepúlveda, Pablo Rebollo, Paloma López-Laiz, Jorge Maurino, Nieves Téllez Lara

**Affiliations:** ^1^Centre d’Esclerosi Múltiple de Catalunya (Cemcat), Hospital Universitari Vall d’Hebron, Universitat Autonoma de Barcelona, Barcelona, Spain; ^2^Medical Department, Roche Farma, Madrid, Spain; ^3^Department of Neurology, Hospital Universitario Fundación Jiménez Díaz, Madrid, Spain; ^4^Department of Neurology, Hospital Universitario Virgen del Rocío, Seville, Spain; ^5^Department of Neurology, Hospital Universitario Cruces, Bilbao, Spain; ^6^Department of Neurology, Hospital Álvaro Cunqueiro, Vigo, Spain; ^7^Department of Neurology, Hospital Clínic de Barcelona, Barcelona, Spain; ^8^IQVIA, Madrid, Spain; ^9^Department of Neurology, Hospital Clínico Universitario de Valladolid, Valladolid, Spain

**Keywords:** neuromyelitis optica, therapeutic inertia, severe disease, shared decision-making, high-efficacy treatments

## Abstract

**Introduction and objective:**

Limited information is available on how neurologists make therapeutic decisions in neuromyelitis optica spectrum disorder (NMOSD), especially when new treatments with different mechanisms of action, administration, and safety profile are being approved. Decision-making can be complex under this uncertainty and may lead to therapeutic inertia (TI), which refers to lack of treatment initiation or intensification when therapeutic goals are not met. The study aim was to assess neurologists’ TI in NMOSD.

**Methods:**

An online, cross-sectional study was conducted in collaboration with the Spanish Society of Neurology. Neurologists answered a survey composed of demographic characteristics, professional background, and behavioral traits. TI was defined as the lack of initiation or intensification with high-efficacy treatments when there is evidence of disease activity and was assessed through five NMOSD aquaporin-4 positive (AQP4+) simulated case scenarios. A multivariate logistic regression analysis was used to determine the association between neurologists’ characteristics and TI.

**Results:**

A total of 78 neurologists were included (median interquartile range [IQR] age: 36.0 [29.0–46.0] years, 55.1% male, median [IQR] experience managing demyelinating conditions was 5.2 [3.0–11.1] years). The majority of participants were general neurologists (59.0%) attending a median (IQR) of 5.0 NMOSD patients (3.0–12.0) annually. Thirty participants (38.5%) were classified as having TI. Working in a low complexity hospital and giving high importance to patient’s tolerability/safety when choosing a treatment were predictors of TI.

**Conclusion:**

TI is a common phenomenon among neurologists managing NMOSD AQP4+. Identifying TI and implementing specific intervention strategies may be critical to improving therapeutic decisions and patient care.

## 1 Introduction

Neuromyelitis optica spectrum disorder (NMOSD) is a chronic and devastating autoimmune disease of the central nervous system characterized by inflammatory lesions mainly of the optic nerve and the spinal cord (1). Despite being considered as a rare disease (2), it is a serious condition, as permanent disability or even death are driven by relapses (3–5). Thus, the main therapeutic goal for patients with NMOSD is to reduce the frequency and severity of these episodes with the aim of avoiding long-term disability accumulation (6, 7).

Historically, NMOSD has been treated with off-label treatments, mainly immunosuppressants (ISTs), oral corticosteroids (OCs) or rituximab. However, in recent years, targeted high-efficacy therapies with a positive safety profile have been approved for its management (8). These new treatments have broadened the landscape of available options, but in turn may have complicated the decision-making process (9, 10). A consequence of this difficulty might be the presence of therapeutic inertia (TI) in decision-making, defined as the lack of treatment initiation or intensification when therapeutic goals are not met (11). In fact, TI has been shown to be present in more than half and up to 96% of treatment decisions in a demyelinating disease with multiple treatment choices such as multiple sclerosis (MS) (12). Several factors have been related to TI prevalence in MS, some influenced by neurologist’s professional experience, such as a lower volume of patients, not being an MS specialist, and fewer years of practice; and others by personality-related traits, namely an aversion to ambiguity and lower tolerance to uncertainty (13). However, what might be influencing TI is the complexity of decisions in MS due to the increasing availability of numerous different treatments and the limited training physicians receive in terms of decision-making processes and risk management in complex environments with multiple options and uncertainty (10). Furthermore, complicated treatment monitoring and fear of side effects have been related to TI as well (10, 14).

In a varying context in NMOSD aquaporin-4 positive (AQP4+) with a broadened therapeutic landscape, the lack of head-to-head trials to facilitate decisions, and no clear management guidelines, understanding the decision-making process and its contributors may be important to provide information on how these decisions are made and reach the best patient care approach. Thus, the aim of the present study was to assess the prevalence of TI and associated factors in the clinical decisions of neurologists caring for patients with NMOSD in Spain.

## 2 Methods

### 2.1 Study design and participants

PREFERENCES-NMOSD was an online, non-interventional, cross-sectional, and exploratory study in collaboration with the Spanish Society of Neurology (SEN). From June 16th to September 27th, 2022, neurologists were invited to participate and proactively answered an online survey sent by email to provide details about their demographic characteristics, professional background, and behavioral traits. Being actively involved in the management of NMOSD patients at the time of the study was set as an inclusion criteria. The study was approved by the research ethics board of Hospital Universitario Clínico San Carlos, Madrid, Spain and performed in accordance with the 1964 Helsinki Declaration and its later amendments. All participants provided written informed consent.

### 2.2 Outcome measures

We explored the prevalence of TI through five simulated NMOSD AQP4+ clinical practice case scenarios designed by the research team (Supplementary material). Treatment options for case scenarios were presented unbranded (Drug A-F) as in Table 1, mimicking the current available landscape of treatments for NMOSD. For its creation, we used the available scientific evidence of efficacy, safety, and route and frequency of administration from literature on clinical trials and off-label treatments (15–20).

**Table 1:** Hypothetical treatments for case scenarios.

	Efficacy (reduction of risk of new relapses)	Mild but frequent adverse effect (20–30%)	Serious but rare adverse effect (1–5%)
DRUG A Oral: 1 time/day	Approx 30%	Leukopenia Infections	Hepatotoxicity and malignancies
DRUG B Oral: 2 times/day	Approx 50%	Infections Anemia Leukopenia Headache Gastrointestinal disorders	Lymphoproliferative and dermatological malignancies. Predisposition to infections including PML
DRUG C IV: every 6 months	Approx 70%	Infusion reactions (itching, throat irritation, tachycardia, fever, shortness of breath) Urinary tract infections Respiratory tract infections	Serious infections
DRUG D SC: every 4 weeks	Approx 80%	Upper respiratory tract infections Headache Nasopharyngitis	Long-term risk of hyperlipidemia
DRUG E IV: every 6 months	Approx 80%	Urinary tract infections Arthralgia	Serious infections
DRUG F IV: every 2 weeks	Approx 90%	Urinary tract infections Headache	Risk of infection by encapsulated bacteria

Approx, approximately; IV, intravenous; PML, progressive multifocal leukoencephalopathy; SC, subcutaneous.

Due to the severity of NMOSD relapses and disability accumulation, we used a stricter definition of therapeutic inertia, described in our study as the lack of pursuit of high-efficacy treatments when there is evidence of disease activity (based on clinical course and neuroimaging markers) (21). For the same reason, we established the term therapeutic error (TE) for case scenarios where, despite evidence of disease activity, the decision was to deescalate to a lower efficacy treatment (cases 3 and 4). In our study, high-efficacy treatments were considered those having more than 70% of efficacy in reduction of risk of new relapses (22), that is, Drugs C- F in Table 1.

The Evidence-Based Practice Attitude Scale (EBPAS), Provider Decision Process Assessment Instrument (PDPAI), Jefferson Scale of Physician Empathy (JSPE), Regret Intensity Scale (RIS-10), General Risk Propensity Scale (GRiPS), and Reasons for Treatment Selection Questionnaire (RTSQ) were used to gather information on neurologists’ attitudes to innovation, decision-making comfort, empathy, care-related regret, risk attitude, and treatment decision, respectively.

The EBPAS is a validated questionnaire assessing the attitude toward the adoption of new treatments, interventions, and practices among healthcare professionals with 15 items divided into four subscales: Requirements, Appeal, Opening, and Divergence. Scores range from 0 (not at all) to 4 (to a very great extent), with higher scores indicating a more positive attitude toward innovation, except for Divergence (reversed) (23).

The PDPAI is a 12-item questionnaire measuring healthcare professional comfort regarding a medical decision. In our study, neurologists were asked to think about their contentment when making medical decisions in NMOSD. Each item is scored using a 5-point Likert scale ranging from 1 (strongly agree) to 5 (strongly disagree). Total score ranged from 12 to 60 as a result of adding each item response after reversing some of the items. Lower scores imply a better decision-making process (24).

The JSPE assesses physicians’ empathy using 20 items scored on a 7-point Likert scale that ranges from 1 (strongly disagree) to 7 (strongly agree). Total score ranged from 20 to 140 after adding each item and reversing some of them. Higher scores are associated with a higher degree of empathy (25).

The RIS-10 assesses the intensity of care-related regret at the time of measurement caused by a past decision or event occurring up to 5 years earlier. Items are scored using a 5-point Likert scale ranging from 1 (strongly disagree) to 5 (strongly agree), with higher scores indicating higher intensity of regret (26).

The GRiPS is an 8-item questionnaire measuring the attitude to risk. Each item is assessed on a 5-point Likert scale from 1 (strongly disagree) to 5 (strongly agree), with higher scores meaning higher risk-taking behavior (27).

The RTSQ evaluates the reasons leading to a treatment decision with 22 items, which are grouped in 5 categories based on knowledge of recent empirical findings (theoretical), personal experiences (experiential), patient tolerance (situational), anticipation about the course of a therapy, and patient preferences (interactional). Each item is scored using a 5-point Likert scale ranging from 0 (irrelevant) to 4 (decisive) (28).

### 2.3 Statistical analyses

Demographic and clinical characteristics were summarized using frequencies (percentages) and mean (standard deviations). TI was considered to be present if there was at least one incorrect response based on the definition, and this was used to calculate the sample prevalence of TI. TE was present when the participant selected the second answer (Switch to drug B) in case scenarios 3 and 4.

Neurologists’ demographic, professional, and behavioral characteristics were compared among groups with presence/absence of TI using independent t-test for continuous variables and Chi-square test for categorical variables. Values of *p* < 0.05 were considered significant.

Demographic, professional and behavioral factors associated with the presence of TI were analyzed using univariate (association of the dichotomous outcome variable – presence of TI – with one predictor factor) and multivariate logistic regressions. Variables with value of p less than 0.1 in the univariate analysis were included in the multivariate model. Categorical variables were considered significant in the univariate model when at least one of the categories resulted significant. All variables (except type of hospital) were included as continuous. Odds ratios with 95% confidence intervals were derived.

## 3 Results

### 3.1 Demographic and professional characteristics of the sample

A total of 1,400 neurologists were invited to participate through the Spanish Society of Neurology. Of those, 154 agreed to participate (11%), and 117 met selection criteria (8.4%). Finally, a total of 78 neurologists (5.6%) completed the therapeutic inertia assessment and were included in the study, representing a 13% of Spanish neurologists who potentially treat demyelinating conditions. The median [interquartile range (IQR)] age was 36.0 (29.0–46.0) years, 55.1% were male, and the median (IQR) time of experience managing demyelinating conditions was 5.2 (3.0–11.1) years. The majority of participants were general neurologists (59.0%) attending a median (IQR) of 5.0 (3.0–12.0) NMOSD patients annually. Demographic, professional, and other characteristics of the sample are shown in Table 2.

**Table 2:** Demographic and professional characteristics.

Characteristic	*N* = 78
Age, years, median (IQR)	36.0 (29.0–46.0)
Sex (male), *n* (%)	43 (55.1)
Years of experience as neurologist, median (IQR)	9.1 (4.2–15.0)
Years of experience managing MS/NMOSD patients, median (IQR)	5.2 (3.0–11.1)
Degree of specialization, *n* (%)	
Subspecialized in MS/NMOSD	32 (41.0)
Type of hospital*, *n* (%)	
Regional	0 (0.0)
Basic general or low complexity	8 (10.3)
Medium complexity	22 (28.2)
Large	14 (17.9)
Complex or referral	24 (30.8)
CSUR	10 (12.8)
Number of MS patients managed by responding neurologist in 1 week, median (IQR)	15.0 (4.0–25.0)
Number of NMOSD patients managed by responding neurologist in 1 year, median (IQR)	5.0 (3.0–12.0)
Number of neurologists managing MS/NMOSD patients with responding neurologist, median (IQR)	4.0 (3.0–5.0)
Participation in NMOSD clinical trials, yes, *n* (%)	4 (5.1)
Author or co-author of scientific manuscripts in peer-reviewed journals in last 3 years, yes, *n* (%)	39 (50.0)
Attended ECTRIMS congress either in person or virtually in last 3 years, yes, *n* (%)	45 (57.7)
EBPAS, mean (SD)	2.9 (0.4)
GRiPS, mean (SD)	18.8 (6.4)^a^
RIS-10, mean (SD)	20.2 (8.4)
RTSQ, mean (SD)	
Theoretical knowledge	2.3 (0.5)
Experiential knowledge	2.3 (0.5)
Situational knowledge	2.3 (0.6)
Attitudes and anticipations about the course of therapy	3.0 (0.5)
Interactional knowledge	2.1 (0.6)
PDPAI, mean (SD)	30.1 (6.4)
JSPE, mean (SD)	114.9 (12.3)

*Regional hospital (<150 beds), basic general hospital (<200 beds), medium complexity area hospital (average 500 beds, >50 MIR physicians), large hospital (at least 4 high complexity services and > 160 MIR physicians), complex or referral hospital (>680 physicians, 300 MIR physicians) and CSUR (Center accredited as a national service for the MS care process by the Spanish Ministry of Health). ^a^*N* = 77. EBPAS, Evidence-Based Practice Attitude Scale; ECTRIMS, European Committee for Treatment and Research in Multiple Sclerosis; GRiPS, General Risk Propensity Scale; IQR, interquartile range; JSPE, Jefferson Scale of Physician Empathy; MIR, Resident Medical Intern; MS, multiple sclerosis; NMOSD, neuromyelitis optica spectrum disorder; PDPAI, Provider Decision Process Assessment Instrument; RIS-10, Regret Intensity Scale; RTSQ, Reasons for Treatment Selection Questionnaire; SD, standard deviation.

### 3.2 Descriptive data of therapeutic inertia

Thirty participants (38.5%) were classified as having TI in at least one of the presented case scenarios, and 10 participants (12.8%) in 2 or more responses (Table 3). Therapeutic error was found in seven participants (9%) in at least one of the case scenarios (cases 3 and 4). Using the classical definition of TI (not starting or escalating when therapeutic goals are not achieved) led to a similar percentage (32.1%, *n* = 25) of results.

**Table 3:** Therapeutic inertia related to case scenarios.

		Total
CASE 1	Total	78 (100.0%)
	Do not change treatment*	1 (1.3%)
	Switch to drug B*	10 (12.8%)
	Switch to drug C	10 (12.8%)
	Switch to drug D	57 (73.1%)
CASE 2	Total	78 (100.0%)
	Do not change treatment, re-administer when appropriate*	2 (2.6%)
	Switch to drug D	17 (21.8%)
	Switch to drug E	13 (16.7%)
	Switch to drug F	46 (59.0%)
CASE 3	Total	78 (100.0%)
	Do not change the treatment and wait for 1 year follow-up evaluation*	15 (19.2%)
	Switch to drug B*^†^	5 (6.4%)
	Switch to drug D	52 (66.7%)
	Switch to drug F	6 (7.7%)
CASE 4	Total	78 (100.0%)
	Maintain treatment and follow-up at 6 months*	7 (9.0%)
	Switch to drug B*^†^	2 (2.6%)
	Switch to drug D	31 (39.7%)
	Switch to drug E	16 (20.5%)
	Switch to drug F	22 (28.2%)
CASE 5	Total	78 (100.0%)
	No treatment initiation*	1 (1.3%)
	Treatment initiation on drug B*	3 (3.8%)
	Treatment initiation on drug C	15 (19.2%)
	Treatment initiation on drug D	28 (35.9%)
	Treatment initiation on drug F	31 (39.7%)
Number of TI	Total	78 (100.0%)
	0	48 (61.5%)
	1	20 (25.6%)
	2	5 (6.4%)
	3	4 (5.1%)
	4	1 (1.3%)
At least one TI response	Total	78 (100.0%)
	No	48 (61.5%)
	Yes	30 (38.5%)

*Answers considered therapeutic inertia based on the definition. ^†^Answers considered therapeutic error based on the definition. TI, therapeutic inertia.

### 3.3 Associations between neurologists’ features and therapeutic inertia

Participants with TI showed a lower degree of empathy (JSPE scale; *p* = 0.0005) and perceived research-based interventions as not clinically useful and less important than clinical experience in the EBPAS scale (*p* = 0.0014). When choosing a treatment, neurologists with TI gave more importance to ease of use (RTSQ item 9; *p* = 0.0424), patient tolerability/safety (RTSQ item 5; *p* = 0.0057), or made the choice casually (RTSQ item 18; *p* = 0.0128), while giving lower importance to the existence of scientific evidence in favor of treatment in the RTSQ questionnaire (item 2; *p* = 0.0030) (Table 4).

**Table 4:** Associations between demographic, professional and behavioral characteristics and therapeutic inertia (*n* = 78).

Variable	No TI	TI (at least one)	Value of *p*
Age (years), mean (SD)	38.69 (10.22)	37.93 (11.78)	0.7659
Gender, *n* (%)			0.829
Male	26 (54.2%)	17 (56.7%)	
Female	22 (45.8%)	13 (43.3%)	
Years of experience as neurologist, mean (SD)	11.59 (8.45)	10.92 (9.24)	0.7459
Years of experience managing MS/NMOSD patients, median (IQR)	4.2 (3.0; 11.6)	5.8 (2.7; 11.1)	0.9707
Degree of specialization, n (%)			0.1176
Neurologist subspecialized in MS/NMOSD	23 (47.9%)	9 (30.0%)	
General neurologist involved in treating patients with MS/NMOSD	25 (52.1%)	21 (70.0%)	
Type of hospital			0.2353
Regional	0	0	
Basic general or low complexity	3 (6.3%)	5 (16.7%)	
Medium complexity	11 (22.9%)	11 (36.7%)	
Large hospital	9 (18.8%)	5 (16.7%)	
Complex or referral	17 (35.4%)	7 (23.3%)	
CSUR	8 (16.7%)	2 (6.7%)	
Number of MS patients managed by responding neurologist in 1 week, median (IQR)	15.0 (3.0; 23.5)	12.5 (5.0; 30.0)	0.7124
Number of NMOSD patients managed by responding neurologist in 1 year, median (IQR)	5.0 (2.5; 11.0)	5.0 (3.0; 15.0)	0.7536
Number of neurologists managing MS/NMOSD patients with responding neurologist, median (IQR)	3.0 (3.0; 5.0)	4.0 (3.0; 6.0)	0.3579
Participation in NMOSD clinical trials			0.6263
No	46 (95.8%)	28 (93.3%)	
Yes	2 (4.2%)	2 (6.7%)	
Author or co-author of scientific manuscripts in peer-reviewed journals in the last 3 years			0.3519
No	22 (45.8%)	17 (56.7%)	
Yes	26 (54.2%)	13 (43.3%)	
Have you attended the ECTRIMS congress either in person or virtually in the last 3 years?			0.1192
No	17 (35.4%)	16 (53.3%)	
Yes	31 (64.6%)	14 (46.7%)	
EBPAS global score, mean (SD)	2.99 (0.43)	2.84 (0.43)	0.1429
Requirements subscale	2.83 (0.92)	2.78 (0.88)	0.7924
Appeal subscale	2.71 (0.65)	2.80 (0.71)	0.5589
Openness subscale	3.17 (0.70)	3.13 (0.67)	0.7709
Divergence subscale (not reversed)	0.77 (0.56)	1.35 (0.99)	**0.0014**
PDPAI total score, mean (SD)	29.60 (6.42)	30.87 (6.32)	0.398
JSPE total score, mean (SD)	118.63 (9.29)	108.87 (14.24)	**0.0005**
RIS-10 global score, mean (SD)	20.67 (9.21)	19.50 (6.91)	0.5529
GRiPS^a^ total score, mean (SD)	18.38 (6.75)	19.48 (5.74)	0.4634
RTSQ items, mean (SD)			
Theoretical knowledge (item 2)	3.2 (0.6)	2.8 (0.7)	**0.0030**
Theoretical knowledge (item 18)	1.0 (0.9)	1.5 (0.8)	**0.0128**
Theoretical knowledge (item 19)	2.3 (1.0)	2.2 (1.0)	0.6632
Theoretical knowledge (item 22)	2.7 (1.3)	2.4 (1.1)	0.3392
Experiential knowledge (item 1)	2.8 (0.8)	2.8 (0.6)	0.9256
Experiential knowledge (item 9)	2.1 (0.8)	2.5 (0.8)	**0.0424**
Experiential knowledge (item 12)	2.0 (0.8)	2.3 (0.8)	0.1243
Experiential knowledge (item 17)	2.8 (0.7)	2.6 (0.7)	0.4684
Experiential knowledge (item 20)	2.0 (1.0)	1.9 (0.7)	0.6263
Experiential knowledge (item 21)	1.9 (1.0)	1.6 (0.9)	0.2834
Situational knowledge (item 5)	2.2 (1.0)	2.8 (0.7)	**0.0057**
Situational knowledge (item 11)	1.7 (1.0)	1.8 (0.9)	0.523
Situational knowledge (item 13)	2.6 (0.6)	2.7 (0.6)	0.6068
Attitudes and anticipations about the course of therapy (item 3)	3.0 (0.6)	2.8 (0.6)	0.0867
Attitudes and anticipations about the course of therapy (item 7)	2.8 (1.0)	2.8 (0.8)	0.9382
Attitudes and anticipations about the course of therapy (item 10)	3.4 (0.7)	3.2 (0.9)	0.1706
Interactional knowledge (item 4)	2.5 (0.7)	2.6 (0.7)	0.7342
Interactional knowledge (item 6)	2.2 (1.1)	2.5 (0.9)	0.1383
Interactional knowledge (item 8)	1.4 (0.9)	1.6 (0.7)	0.2837
Interactional knowledge (item 14)	2.4 (0.7)	2.5 (1.0)	0.7538
Interactional knowledge (item 15)	1.8 (1.0)	2.1 (0.9)	0.0958
Interactional knowledge (item 16)	2.1 (0.8)	2.2 (0.9)	0.7034

*Regional hospital (<150 beds), basic general hospital (<200 beds), medium complexity area hospital (average 500 beds, >50 MIR physicians), large hospital (at least 4 high complexity services and > 160 MIR physicians), complex or referral hospital (>680 physicians, 300 MIR physicians) and CSUR (Center accredited as a national service for the MS care process by the Spanish Ministry of Health). ^a^*N* = 77. EBPAS, Evidence-Based Practice Attitude Scale; ECTRIMS, European Committee for Treatment and Research in Multiple Sclerosis; GRiPS, General Risk Propensity Scale; IQR, interquartile range; JSPE, Jefferson Scale of Physician Empathy; MIR, Resident Medical Intern; MS, multiple sclerosis; NMOSD, neuromyelitis optica spectrum disorder; PDPAI, Provider Decision Process Assessment Instrument; RIS-10, Regret Intensity Scale; RTSQ, Reasons for Treatment Selection Questionnaire; SD, standard deviation. Bold values represent *p* < 0.05.

When evaluating neurologists’ characteristics predictive of TI, the binary regression univariate model found that working in a low complexity hospital (basic general hospital with less than 200 beds), perceiving research-based interventions as not clinically useful in the EBPAS scale, and showing a lower degree of empathy in the JSPE scale were associated to TI. Different items in the RTSQ scale were associated to TI as well, such as giving more importance to ease of use (item 9), patient tolerability/safety (item 5), or patient’s social factors (item 15) when choosing a treatment or making the choice casually (item 18), while giving lower importance to the existence of scientific evidence in favor of treatment (item 2). Finally, the multivariate analysis found that working in a low complexity hospital [OR = 104.394 (95% CI 1.67–999.99), *p* = 0.0276] and giving high importance to patient tolerability/safety when choosing a treatment [RTSQ item 5; OR = 32.04 (95% CI 3.25–315.57), *p* = 0.0030] were independently associated to the risk of TI.

## 4 Discussion

Understanding the current treatment decision-making process in NMOSD is of significant relevance, as there are few consensus or management guidelines and new therapies have been approved for this condition in the last few years (6, 15, 29, 30). Due to the severity of relapses in this condition, which result in disability accumulation and can even lead to death (83% of patients with partial or no recovery from relapses and mortality rates vary from 3.3–25%) (3–5, 31, 32), we classified TI as not pursuing high-efficacy treatments when therapeutic goals are not met. We found that TI is a common phenomenon in the management of NMOSD patients, observed in more than one-third of participating neurologists. Factors associated with TI were working in a low complexity hospital and giving higher importance to patient tolerability/safety when choosing a treatment.

The management of patients with NMOSD has become more challenging with the approval of the first new therapies. Despite these advances in the last few years, there is little information on treatment decisions including new agents. In this study, we specifically assessed TI in NMOSD with unbranded treatments mimicking the available ones. Min et al. performed a clinical record review in six countries worldwide and found that relapses in NMOSD do not always lead to immediate initiation of maintenance therapy or a switch from off-label OC/ISTs to higher efficacy monoclonal antibodies (33). For newly diagnosed patients, they found that 47% did not receive maintenance treatment within 2 months of diagnosis for reasons such as disease stability, patient refusal and cost/access restrictions, being the severity of relapse a factor related to immediate treatment initiation (33). They also found that 32% of neurologists strongly agreed on initiating OC/IST as first-line therapy in patients with moderate-to-severe symptoms, and 38% agreed if symptoms were mild. When exploring treatment escalation, lack of efficacy, relapse severity and insufficient recovery from relapse drove 54% of all switches (33). However, almost half of the patients most recently seen by the participating neurologists had a relapse and did not change their treatment, mainly for reasons such as relapse mildness, patient stability, or receiving good treatment already (33). Furthermore, a proportion of 42.5% of patients were switched between different OC/ISTs when they had a relapse instead of escalating to a high-efficacy monoclonal antibody, even though half of those relapses were moderate-to-severe in nature (33). This would imply a similar-to-higher percentage of TI than in our study, although new agents were not available in all countries at the time of survey completion. Similar to our results, Thon et al. found that 40% of neuroimmunologists (*n* = 10/25) would switch none or up to 25% of their patients to one of the novel NMOSD treatments following a relapse (34). The authors hypothesized that the reasons behind this decision could be due to insurance and cost-related barriers (34). However, these factors did not apply in our study as treatments were unbranded and only efficacy/safety data was provided to respondents.

Another survey about treatment choices performed in Korea before new treatments’ approval found that ISTs were prescribed by 85% of the 27 participating neurologists as first-line therapies, and 70% of them would switch to rituximab as second-line therapy if there was a relapse (35). Participants treating a higher number of NMOSD patients annually were more likely to prescribe rituximab as second-line therapy (35). Similarly, a higher volume of patients managed by the responding neurologist has been associated with lower TI in other studies in MS, as well as more years of experience or being an MS specialist (12, 13). However, in our study, the only professional characteristic found as a predictor of TI was working in a low complexity hospital. Additionally, although we found a higher prevalence of TI among neurologists with a lower degree of empathy, and those who gave higher importance to treatment ease of use and their own experience rather than research or scientific evidence, the only predictor of TI in the multivariate analysis in this matter was giving higher importance to patient tolerability/safety when choosing a treatment. We hypothesized that all these factors could be strategies adopted by the respondent in an attempt to reduce the ambiguity and risk-aversion that has been related with TI in previous studies (13).

Uncertainty due to the limited experience with new agents and their differences, the rarity of the disease, the lack of clinical management guidelines including the increasing therapeutic options, together with long experience with off-label treatments, might be affecting the decision-making process (36), giving importance to relapse severity or lesion location as a driver of switches (33, 37). However, there is no clear consensus on how relapse severity is defined in clinical practice and current mildness does not necessary reflect the same in subsequent relapses, the prevention of all relapses being the therapeutic goal regardless of their severity (6, 7, 38, 39). In fact, patients describe disease stability as the absence of any relapse, since they focus on the impact relapses have on daily life and wellbeing (40, 41). Thus, the relapsing nature of the disease together with the importance of a patient-centered approach with shared decision-making should lead to a shift in the treatment paradigm to early use of high-efficacy therapies when treating this condition, as it is evolving in the MS field (42). This is a matter of importance in NMOSD due to the severity of relapses, poor recovery, and associated disability accumulation (5, 22, 39, 43). New therapeutic landscapes might facilitate reaching this ambition, as recent studies have shown an increase in the use of high-efficacy monoclonal antibodies with better outcomes at the expense of IST/OCs (7, 22, 44). However, as shown in this study, there is still a need to improve therapeutic decisions in order to reduce the prevalence of TI and its magnitude, as new highly effective therapies may be an advantage, but the choice between multiple options might lead to suboptimal decisions. For this reason, it could be helpful to implement continuous updated medical education and training with innovative therapeutic interventions that facilitate the decision-making process, with the aim to achieve better patient outcomes (45–47). As NMOSD is a rare disease, patients also could benefit from being treated at referral centers, where continuous training and a greater number of patients assisted is usually more common, thus providing the center with higher expertise and specialization.

Several limitations should be mentioned. First, the cross-sectional design did not allow us to assess changes or causal relationships in neurologists’ TI over time, as the study consisted of a single online survey. Furthermore, we acknowledge it would had been better to perform a prospective or two stage study with training implementation and subsequent measurement of results. Second, there could be potential selection bias in including people with a greater interest in collaborating or who are actively involved with the society. Third, case scenarios did not include all the details (e.g., MRI lesions) or all patient situations, such as adolescents or patients with concurrent autoimmune conditions, but they do contain the minimal information in order to make proper decisions and not develop cognitive fatigue, which could affect the results. Fourth, the study was only performed in Spain, and generalizability of results might not apply to other countries. However, country-specific factors that could affect decisions such as price or insurance cover were removed from the case scenarios. Fifth, we only explored TI in NMOSD AQP4+, without including seronegative patients, as new treatments are approved only in seropositive population. Future studies should be performed to fill these gaps.

In conclusion, TI was detected in 4 out of 10 participating neurologists when initiating or switching treatments in NMOSD AQP4+, a severe condition with accumulating disability led by potentially devastating relapses. This study uncovers the need to challenge the therapeutic status quo in NMOSD, pursuing high-efficacy treatments from the start, and developing specific intervention strategies to ensure optimal therapeutic decisions and patient care. Future longitudinal studies should investigate this matter and strategies to reduce TI in NMOSD, including patient perspectives.

## Data availability statement

Qualified researchers may request access to individual level data through the clinical study data request platform (https://vivli.org/). Further details on Roche’s criteria for eligible studies are available here (https://vivli.org/members/ourmembers/). For further details on Roche’s Global Policy on the Sharing of Clinical Information and how to request access to related clinical study documents, see here https://www.roche.com/innovation/process/clinical-trials/data-sharing.

## Ethics statement

The studies involving humans were approved by the research ethics board of Hospital Universitario Clínico San Carlos, Madrid, Spain. The studies were conducted in accordance with the local legislation and institutional requirements. The participants provided their written informed consent to participate in this study.

## Author contributions

ÁC-C: Conceptualization, Data curation, Writing – original draft, Writing – review & editing. RG-B: Conceptualization, Writing – original draft, Writing – review & editing. AO: Data curation, Writing – review & editing. MD: Data curation, Writing – review & editing. SB: Data curation, Writing – review & editing. MA-V: Data curation, Writing – review & editing. MS: Data curation, Writing – review & editing. PR: Conceptualization, Writing – review & editing. PL-L: Conceptualization, Writing – review & editing. JM: Conceptualization, Writing – original draft, Writing – review & editing. NTL: Conceptualization, Data curation, Writing – original draft, Writing – review & editing.
