# Supplementary material for: Therapeutic inertia in the management of neuromyelitis optica spectrum disorder
Source: Front Neurol. 2024 Feb 21;15:1341473. doi: 10.3389/fneur.2024.1341473 (PMC10915282; doi:10.3389/fneur.2024.1341473)
Supplement: Supplementary file 1 [file Data_Sheet_1.docx]

**Supplementary Material**

Simulated case scenarios as presented to participants (answers in bold were considered suboptimal treatment decisions):

**Please answer the following case scenarios as if all drugs were available in your hospital, but do not consider price when choosing an option. All cases represent NMOSD AQP4 seropositive patients.**

**Case 1:** 43-year-old woman diagnosed with NMOSD 5 years ago, with no further relapse since that date. She was treated with drug A and her disability was moderate (EDSS scale=3) until one month ago when she presented a relapse of optic neuritis in the left eye with impaired vision that has not been recovered.

What would your preference be? Please select one option:

**1. Do not change treatment**

**2. Switch to drug B**

3. Switch to drug C

4. Switch to drug D

**Case 2:** 35-year-old woman diagnosed with NMOSD 2 years ago, with no further relapse since that date. She was treated with drug C and her disability was mild (EDSS scale=2) until 3 months ago when she presented a spinal cord relapse with involvement of three segments which led her to an EDSS of 7. After recovery, her disability has been established at an EDSS of 4.

What would your preference be? Please select one option:

**1. Do not change treatment, re-administer when appropriate.**

2. Switch to drug D

3. Switch to drug E

4. Switch to drug F

**Case 3**: 55-year-old woman diagnosed with NMOSD 10 years ago and currently on treatment with drug C. On her next visit to the clinic, she mentioned that she had been having intractable hiccups for the last month.

What would your preference be? Please select one option:

**1. Do not change the treatment and wait for 1 year follow-up evaluation**

**2. Switch to drug B**

3. Switch to drug D

4. Switch to drug F

**Case 4**: 43-year-old male diagnosed 2 years ago with NMOSD due to a spinal cord relapse with paraparesis that required hospital admission. After treatment with plasmapheresis, he recovered with an EDSS=2. He has been treated with drug C since then and 2 months ago he presented a relapse of optic neuritis with vision loss that he has recovered.

What would your preference be? Please select one option:

**1. Maintain treatment and follow up at 6 months**

**2. Switch to drug B**

3. Switch to drug D

4. Switch to drug E

5. Switch to drug F

**Case 5**: 27-year-old woman recently diagnosed with NMOSD due to a relapse of optic neuritis with chiasm involvement and AQP4 antibodies. She has elevated serum GFAP and homocysteine levels, considered to be a bad prognostic factor.

What would your preference be? Please select one option:

**1. No treatment initiation**

**2. Treatment initiation on drug B**

3. Treatment initiation on drug C

4. Treatment initiation on drug D

5. Treatment initiation on drug F
